# Effects of reducing screen media use on mental health, sleep, physical activity, heart rate variability, and behavioral patterns in peergroups of young people: a study protocol for a cluster-randomized controlled trial

**DOI:** 10.1186/s40359-026-04827-5

**Published:** 2026-05-28

**Authors:** Jesper Schmidt-Persson, Anne Kaer Gejl, Kasper Berg Philipsen, Maja Sulstad Johansen, Jonas Mathiesen, Rachael Taylor, Trine Flensborg-Madsen, Peter Lund Kristensen, Anders Grøntved

**Affiliations:** 1 Department of Sports Science and Clinical Biomechanics, Research Unit for Exercise Epidemiology, Centre of Research in Childhood Health, University of Southern Denmark, Odense M, Denmark; 2 Department of Medicine, University of Otago, Dunedin, New Zealand; 3 Child Psychological Development, National Institute of Public Health, Faculty of Health Sciences, University of Southern Denmark, Copenhagen, Denmark

**Keywords:** Screen time, Digital media, Social interaction, Peer group, Wearable device, Mental health, Physiological stress, Physical activity, Sleep

## Abstract

**Background:**

Digital media has become deeply embedded in the daily lives of young people, raising concerns about potential effects on mental health, sleep, and physical activity. Although observational studies suggest associations between extensive screen media use and adverse health outcomes, causal evidence remains limited due to confounding and reliance on self-reported measures. Most experimental studies have also focused on individual-level interventions and adult populations. In addition, little is known about how screen media use may influence social dynamics among peers. The PeerScreen trial aims to investigate the effects of reducing screen media use on mental health, sleep, physical activity, heart rate variability, and behavioral patterns among adolescents and young adults within naturally occurring peer groups.

**Methods:**

The PeerScreen trial is a parallel-group cluster-randomized controlled trial in which peer groups of adolescents and young adults aged 13–24 years are randomized to either a four-week screen media reduction intervention or a control condition. Participants in the intervention group will be instructed to reduce smartphone use to one hour per day and television, computer, and tablet use to 14 h per week, and leave screen media devices outside the bedroom. We plan to recruit 80 peer groups including 2–6 peers. Data collection combines questionnaires on mental health and well-being, daily reports of mood, sleepiness, and social interactions, and objective monitoring of screen media use, sleep, physical activity, and heart rate variability using a custom application and a wearable device. Bluetooth beacon data will be used to objectively assess social interactions and geolocation data will be used to add context to the observed interactions.

**Discussion:**

This study will provide detailed experimental evidence on the effects of reducing screen media use among young people in a real-world peer context and may advance understanding of both individual and social mechanisms linking screen media use to well-being and behavioral measures.

**Trial registration:**

ClinicalTrials.gov: NCT07531290 Date of registration: 2026-04-15.

**Supplementary Information:**

The online version contains supplementary material available at 10.1186/s40359-026-04827-5.

## Introduction

Over the past two decades, digital media has become deeply embedded in the daily routines of young people, raising concerns about their potential impact on health and development. Young people now spend a substantial and increasing portion of their daily time engaging with screen-based electronic media devices [1, 2]. In particular, the use of portable technologies such as smartphones and tablets has grown rapidly over recent decades, accompanied by a sharp rise in the use of social networking platforms [2]. This widespread adoption of digital technologies has not only increased the volume of screen media use, but also fundamentally changed how young people entertain themselves, communicate, and interact with peers, family members, and even professional contacts [3].

Notably, this increase in digital engagement, especially through smartphones, has coincided with a marked rise in mental health issues among young people, prompting growing concern about a possible link between these trends [4, 5]. Systematic reviews support the indication of a relationship between screen media use and adverse mental health outcomes such as depression and stress in young people [6, 7]. Detrimental association between screen media use (particularly around bedtime) and sleep outcomes in youth have also been reported [8]. Some research also indicate that screen media use may pose a significant barrier to physical activity [9], exacerbating concerns about sedentary behaviors and their associated health risks. However, existing research is largely limited by potential reverse causation bias, uncontrolled confounding, and reliance on self-reported screen media use. The possibility of reverse causation, such as poor mental health prompting increased screen use or late bedtimes leading to more screen exposure, adds complexity to the issue and underscores the need for experimental studies to clarify potential causal relationships.

The growing amount of experimental evidence assessing the impact of reducing screen media use (particularly social media) often focus on very short digital detox studies among young adults where participants are randomized on an individual level [10, 11]. Although these recent experimental studies have aimed to address the methodological limitations from observational studies, they often rely on self-reported measures of compliance to the screen use reductions. This remains a key limitation because substantial discrepancies have been observed between self-reported and objective measures screen media use [12, 13]. Furthermore, most existing experimental studies focus narrowly, either on a single device such as smartphones [14–16], on social media use broadly [17–26], or on one specific platform such as Facebook or Instagram [27, 28]. This narrow focus may limit ecological validity, as recreational screen use typically spans multiple devices and platforms, and may also increase the likelihood of behavioral substitution across screen activities [18]. Importantly, if screen use affects mental health partly by displacing time from sleep or face-to-face interactions with friends and family, the total amount and timing of recreational screen use may be at least as important as the specific device or platform involved. Existing interventions have rarely addressed these mechanisms simultaneously within a broader effort to examine causal hypotheses of possible problematic recreational screen use. Additionally, adolescents remain markedly underrepresented in the literature, raising concerns about the generalizability of findings to this key developmental group.

Despite growing attention to the individual effects of screen media use, little is known about how it influences social group dynamics among peers, as it is typically studied as an individual behavior. However, it increasingly shapes how young people engage with each other in everyday social contexts [29]. Although screen-based interactions can facilitate social connection and relations, it may not provide the same social benefits as face-to-face interaction. By displacing time that could otherwise be spent together in person, screen media use may interfere with the quantity of in-person social interactions. In addition, the quality of social interactions through screen based devices may be lower, potentially weakening social connectedness and disrupting the natural rhythm of shared social experiences [30, 31]. Furthermore, a potential consequence is a reduction in interpersonal synchrony which is the spontaneous alignment of movements, emotional expressions, or physiological responses (e.g. heart rate variability) between individuals. Interpersonal synchrony has been recognized as a core component of prosocial behavior and a biologically rooted mechanism for fostering trust, empathy, and social bonding [32]. As such, disruptions to social interactions and interpersonal synchrony may represent a key mechanism through which increased screen media use contributes to declining well-being in youth.

Therefore, the aim of the PeerScreen trial is to rigorously investigate the effects of reducing screen media use and screen-related behaviors hypothesized to influence mental health, social interactions, sleep, physical activity, heart rate variability, and individual behavioral patterns within peer groups of young people (13–24 years of age) in a cluster-randomized controlled trial. Within the peer-group context, we also seek to explore the extent to which reducing screen media use affects interpersonal synchrony within peer groups.

### Objectives

I. To investigate the effects of recreational screen media use restriction on multiple mental health related outcomes, including self-reported well-being, life-satisfaction, and social connectedness, as well as loneliness, stress, symptoms of depression and anxiety, problematic smartphone use, and objectively assessed social interactions.
II. To investigate the effects of screen media use restriction on self-reported stress, mood, sleepiness, and social interactions assessed through daily subjective reports.
III. To investigate the effects of screen media use restriction on parameters of sleep, including quality, duration, bed- and wake-up times, and physical activity parameters, and heart rate variability as a measure of the autonomic nervous system (ANS) functioning.
IV. To explore interpersonal synchrony within peer groups by conducting a detailed sequential analysis of combined data on physical behaviors, social interactions, and geolocation to examine how reducing screen media use affects peer-group dynamics.
V. To exploratorily identify distinct baseline patterns of multi-device screen media use and examine whether these patterns modify the effects of the intervention on mental well-being and related outcomes, and to further explore how intervention-induced changes in screen-use patterns are associated with changes in these outcomes.

### Hypotheses

I. We hypothesize that participants in the intervention group will show greater self-reported mental well-being, life-satisfaction, social connectedness, as well as more time spent with social interactions compared to controls. We further hypothesize greater reductions in loneliness, stress, symptoms of depression and anxiety, and problematic smartphone use compared to controls.
II. We hypothesize that participants in the intervention group will report lower levels of daily stress, sleepiness, as well as better mood and more social interactions across the intervention period compared to controls.
III. We hypothesize that participants in the intervention group will display longer total sleep time and higher levels of physical activity compared to controls, and report higher sleep quality, and earlier bedtimes, as well as improved ANS functioning compared to controls.
IV. We hypothesize that peer groups in the intervention group will exhibit higher levels of social face-to-face interaction, interpersonal synchrony characterized by more aligned physical behaviors and ANS functioning compared to controls.

## Methods

### Study design

The PeerScreen trial is pre-registered at clinicaltrials.gov (NCT07531290). The study will be carried out as a parallel-group, cluster-randomized controlled trial. After two weeks of baseline assessments, youth peer groups are randomly allocated to either undergo a 4-week screen media reduction (intervention group) or to carry on with screen media use as usual (control group). Youth peer groups will be composed of 2–6 persons with regular in-person social interactions (e.g. classmates, friends from leisure activities, or study groups) to reflect close friendships within peer groups. The rationale for intervening on the peer-group level, rather than on an individual level is guided by evidence that adolescents and young adults are strongly influenced by their peers’ behaviors and social norms within their peer groups [33, 34]. Adolescents’ and young adults’ screen behaviors co-vary with their close friends’ habits and norms, i.e. peers interact online using messaging, gaming, and social media engagement. Randomizing single adolescents to reduce use while their close peers continue as usual would be artificial and misaligned with the causal question motivating this trial: what would mental, physical, and social functioning look like if a young person’s close social environment collectively used less time on screen media devices (as in earlier eras with lower screen engagement). To mimic this counterfactual, the unit of intervention must be the naturally occurring peer group because this preserves ecological validity. Additionally, being a part of a group all undergoing the same intervention (i.e. reducing screen media use) is expected to decrease the participants’ feeling of being singled out because their close peers are undergoing the same condition. The peer-group design is therefore also expected to increase compliance with the intervention by fostering collective motivation and shared commitment to the purpose of the project.

#### Randomization

The random allocation of each peer group will occur after each peer group has completed a 14-day baseline assessment protocol. The randomization will be conducted using a computer-generated block-randomization (block size 2–6) stratified by mean peer group age (</≥ 18 years). In practice, a researcher will log in to an online platform (Randomizer.at, Austria) and click “randomize”, thereby securing allocation concealment.

### Participants

**Fig. 1:**
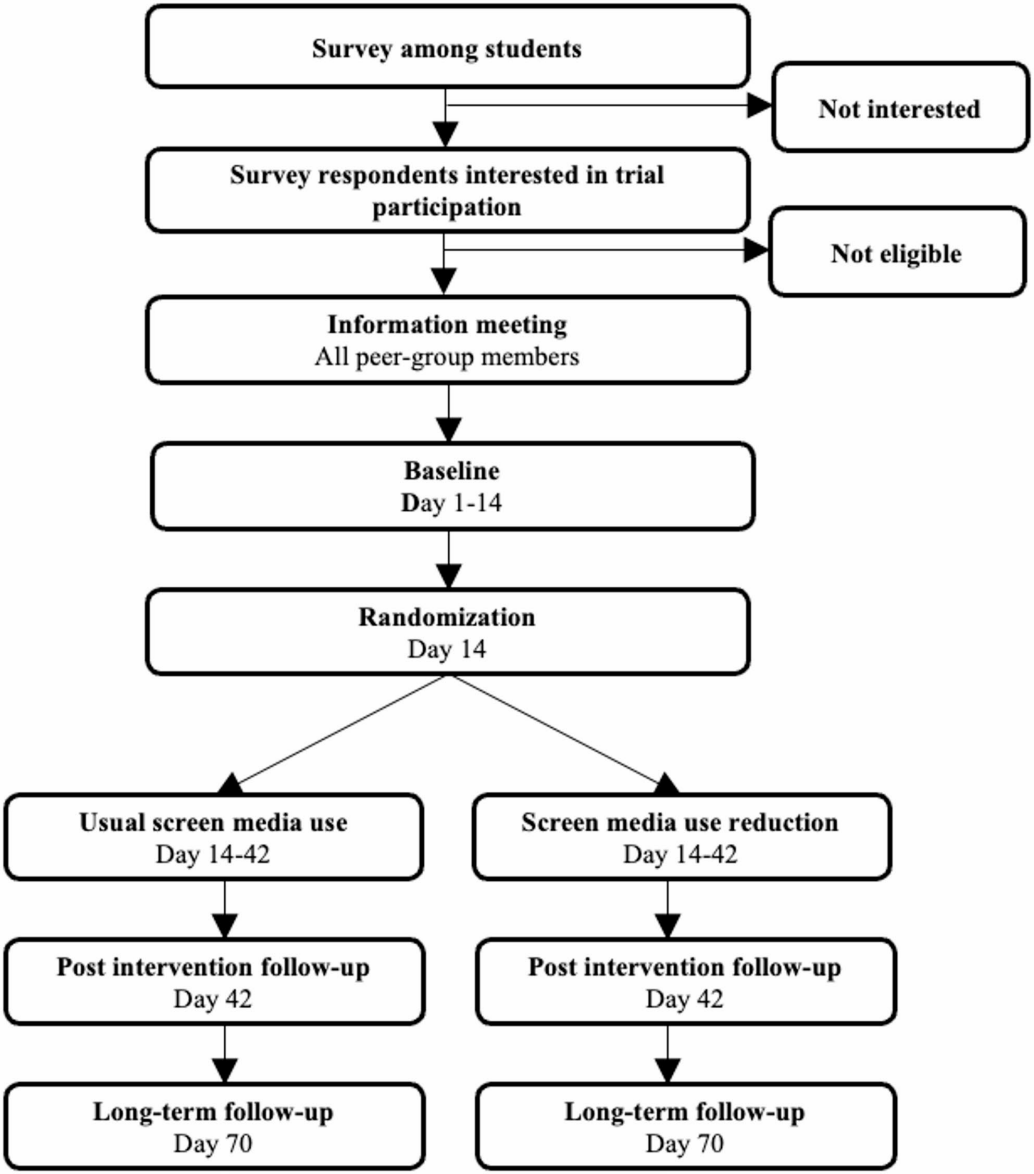
Flow chart

Figure 1 gives an overview of the flow of participants/peer groups in the study. We aim to recruit 80 peer groups of adolescents (13–17 years of age) and young adults (18–24 years of age) via a separate survey study. The survey study is conducted in classes across secondary and higher educational institutions (e.g. secondary schools, high schools, vocational schools, university colleges, and universities) and will describe screen media behaviors among Danish youth. Before administering the survey, the research team will introduce the PeerScreen trial in each class. At the end of the survey, students will be asked whether they are interested in participating in an experimental study and whether the research team may contact them directly for follow-up. Written information about the experiment will be handed out to interested students during the classroom visits, and will also be distributed at the school and through the school’s online platforms together with a registration link after the classroom visits. This will allow students to express their interest at a later stage, if preferred.

Students who express interest (either through the survey or the registration link) will be screened for eligibility in a recruitment interview. The inclusion criteria are as follows: Participants must report having at least 2 h/day of recreational smartphone use (data from the survey study); members of each participant’s household must be willing to support the participant in adhering to their randomly allocated condition (data from recruitment interview); and participants must have enough discretionary leisure time to engage in weekly activities with their peer-group members throughout the study period (verified at the information meeting – see the section “Meeting for peer groups”). The exclusion criteria are: Not owning a smartphone, not having any social media profiles, current participation in other experimental studies, or hospitalization or prescribed sick leave due to a mental illness within the past three months.

Peer groups with regular in-person social interactions will be recruited among eligible individuals who expressed interest, based on existing peer groups, and only one peer group per class will be included at a time to safeguard against intervention contamination. Each individual may only be a part of one peer group. If eligible participants have other naturally occurring peers (persons who they have regular in person social interactions with e.g. during leisure activities), these peers can voluntarily contact the research team on their own and receive study information. If these peers wish to participate, they will be screened for the described eligibility criteria, before they potentially provide their own written informed consent.

If we are unable to recruit enough peer groups through the survey study or the registration link, we will switch to convenience sampling. We will put up posters at secondary and higher educational institutions and make posts on social media sites related to these institutions (e.g. alumni Facebook group or similar) stating that we are looking for peer groups for a research study. Peer-group members voluntarily recruited via convenience sampling will be screened for the same eligibility criteria, before they can potentially provide their own written informed consent.

#### Meetings with peer groups in person

All meetings described in the following section will be conducted in person, with a researcher meeting with the peer-group members.

##### Information meeting

Peer groups (and parents of peer-group members younger than 18 years of age) will be invited to an information meeting. Participants older than 18 years of age are informed that they are welcome to bring a support person to the meeting. A researcher will provide detailed oral information in a quiet setting, ensuring that all participants understand what participation entails. It will be emphasized that taking part in the study is entirely voluntary and that they can withdraw at any time without giving a reason. For peer-groups who remain interested and provide written informed consent at the meeting or at a later point in time, a date for the baseline meeting will be arranged.

##### Baseline meeting (Day 1)

A member of the research team will ensure that written informed consent is obtained for all members of the peer group. A researcher will explain the baseline assessments again. Once the technical equipment has been set up, participants will be handed a paper manual for the technical equipment and an overview of the study period, including contact details for the research staff. Before ending the meeting, all participants will answer the baseline questionnaire on their own.

##### Randomization meeting (Day 14)

A member of the research team will meet with all members of the peer group, and the random group allocation will be performed via the online randomization platform. The group allocation (Intervention/Control) will be revealed live, and the allocated condition is explained in detail to the peer group and written information is provided to make sure they know what to do for the next 4 weeks.

##### Post intervention meeting (Day 42)

A member of the research team meets with all members of the peer group and collects the assessment equipment, makes sure that all questionnaires are completed and reminds participants of the long-term follow-up questionnaire they will receive after 4 weeks (Day 70).

### Conditions

#### Intervention

Participants in peer groups randomly allocated to the intervention will be instructed to reduce several forms of recreational screen media use over a4-week period that are hypothesized to affect mental health and related outcomes. These include high overall recreational screen media use, extensive smartphone use, social media use, solitary screen use, and screen behaviors that may interfere with sleep. Participants will therefore be asked to reduce their recreational screen media use across devices. For smartphone use, participants will be asked to reduce their use to a maximum of 1 h/day. Because social media use will not be permitted on computers or tablets during the intervention, this limit will also encompass participants’ social media use. Research staff will help each participant set up smartphone app limits for the ten most used social media and gaming applications, add a widget to their home screen showing the current amount of time they have spent on their phone, and finally turn off non-essential notifications. For television, computer and tablet use, participants will be asked to reduce their use to 14 h/week (with no specific daily limit), but social media use is not allowed on these devices. We will recommend participants to particularly reduce their solitary screen media use and prioritize co-viewing activities and socially connected gaming with peers and family members during the intervention. Participants will be asked to keep their devices outside the bedroom. To support this, they will be provided with a free alarm clock, as many people use their smartphone to set alarms (see Table 1 for an overview of the intervention components).

**Table 1:** Screen media reduction intervention

	Intervention components
Smartphone use	• Maximum of 1 h/day • Add a widget to their home screen displaying daily smartphone use • Set up smartphone app limits on most used apps (social media and games) • Turn off non-essential smartphone notifications (essential include: calls and text messages)
Television, computer, and tablet use	• Maximum of 14 h/week • No social media use is allowed on these devices and participants are recommended to reduce their solitary screen media use and prioritize co-viewing activities and socially connected gaming with peers and family members
Screen media use and sleep routines	• Leave screen media devices outside the bedroom • Free alarm clock

##### Intervention compliance

The intervention compliance threshold communicated to the participants will be 21 h/week of recreational screen media use (1 h/day smartphone use and 14 h/week of television/computer/tablet use).

#### Control

Participants allocated to the control group will be asked not to change their use of screen media devices and continue with their usual screen media behaviors. At the randomization meeting, if a peer group is allocated to the control condition, a researcher will explain the importance of the control group for ensuring the validity of the trial, with the aim of keeping these groups motivated and engaged. Participants in the control group will be offered the screen media reduction intervention after completing the long-term follow-up. Accordingly, they will function as a waitlist control group, although no outcome data will be collected during their subsequent intervention period.

### Data collection

**Fig. 2:**
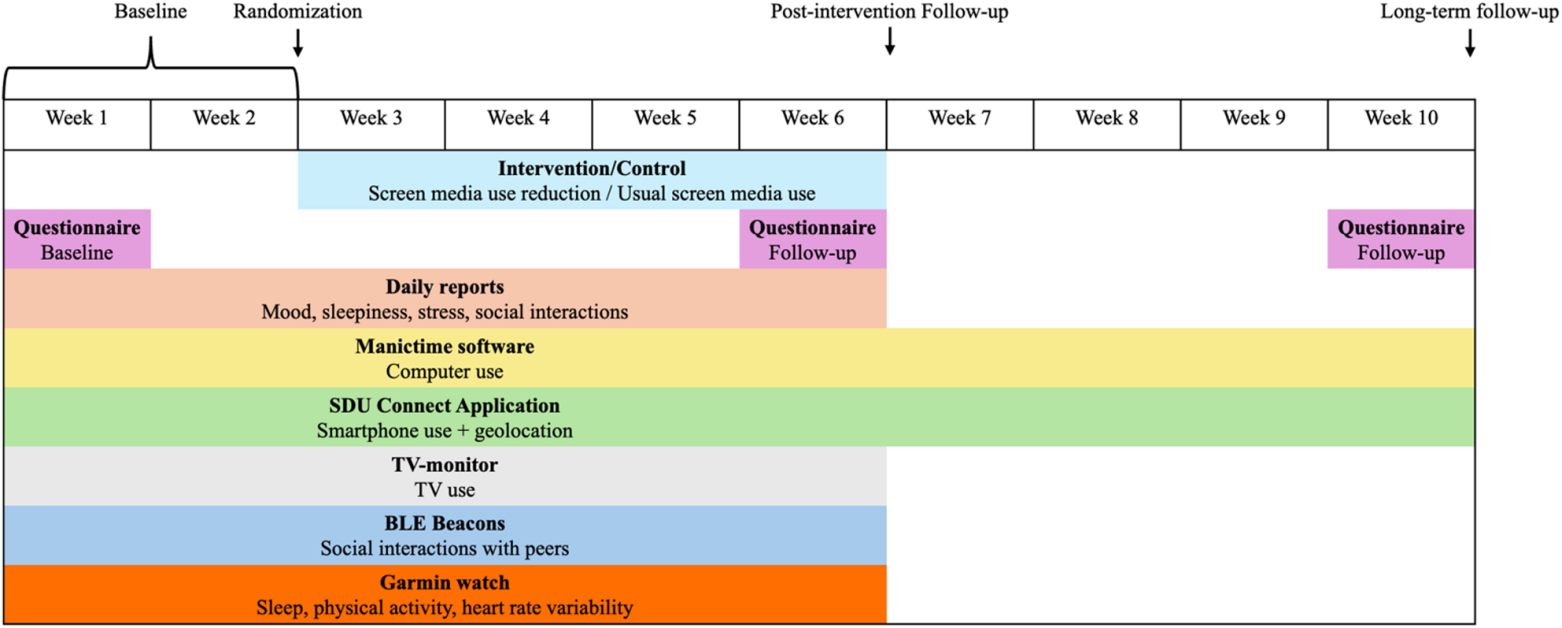
Study overview. Questionnaires at baseline and follow-up include the WEMWBS, Cantril’s ladder for life satisfaction, the 3-item UCLA Loneliness scale, the Generalized Anxiety Disorder 2-item scale, the 13-item Mood and Feelings Questionnaire, the 4-item Cohen’s Perceived Stress Scale, the PROMIS Pediatric Sleep Disturbance Short Form 8a, and the Smartphone Addiction Scale (Short version) described in the section “Subjective assessments”. Daily reports include items for mood, sleepiness, stress and social interactions described in the section “Daily reports”

All participants included will be asked to complete a background questionnaire at the baseline meeting answering questions on birth date, sex, education, work, ethnicity, language, and current accommodation. All participants will also be asked to complete a data collection protocol starting from day 1 (baseline meeting) until day 70. The protocol consists of both subjective and objective assessments (an overview is provided in Fig. 2). The SDU Connect application (developed by Avicenna Research, Canada) will function as the main platform for data collection. The following sections describe the data that will be collected along with the methods and timing of collection.

#### Subjective assessments

Participants will complete the following questionnaires at baseline (day 1), post intervention (day 42), and long-term follow-up (day 70) using the SDU Connect application.

##### Mental well-being

Mental well-being, encompassing positive emotions and psychological functioning, will be assessed by the 14-item Warwick-Edinburgh Mental Wellbeing Scale (WEMWBS) [35]. The scale includes positively worded statements about thoughts, feelings, and functioning over the past two weeks. WEMWBS has been validated among adolescents and adults [36], and has demonstrated responsiveness in intervention studies [37].

##### Life satisfaction

Cantril’s ladder will be used to assess life satisfaction [38]. Participants are asked to rate their life satisfaction by placing themselves on a 10-step ladder, where the top represents the best possible life and the bottom the worst possible life. Cantril’s ladder has been shown to have good validity and reliability among adolescents [39].

##### Loneliness

The UCLA 3-item Loneliness scale will be used to assess participants subjective feelings of loneliness and perceived social isolation [40]. The original 20-item version of the scale has been found to be valid for assessment of loneliness among adolescents and young adults [41]. The 3-item version will be used as it has been found to be an adequate measure of loneliness when compared to the 20-item version [42].

##### Social connectedness

Social connectedness will be assessed using The Friendship Scale, a brief 6-item questionnaire originally developed to measure social isolation and perceived social support [43]. The scale has demonstrated good psychometric properties, including strong correlations with established measures such as the World Health Organization Quality of Life social relationships domain. A Danish translation of the scale has been validated in adults [44].

##### Symptoms of depression

The 13-item scale Mood and Feelings Questionnaire (Short Form) will be used to assess depressive symptoms [45]. Participants are asked to reflect on their experiences over the past two weeks. The Mood and Feelings Questionnaire has been found to be a valid measure of symptoms of depression in adolescents and young adults from the general population [46, 47].

##### Symptoms of anxiety

The Generalized Anxiety Disorder 2-item scale (GAD-2) will be employed to assess core symptoms of anxiety. The GAD-2 assesses symptoms of excessive worry and nervousness and is widely used for brief screening of generalized anxiety in the general population [48]. The GAD-7 has previously been used among young people aged 12–19 years, and GAD-2 consist of the first two items of the 7-item version of GAD [49].

##### Perceived stress

The 4-item version of Cohen’s Perceived Stress Scale measures the degree to which participants find their lives unpredictable, uncontrollable, and overloaded. Cohen’s Perceived Stress Scale is a widely used tool for assessing perceived stress, and the 4-item version has been found to have high internal consistency and reliability, and is significantly correlated with several criterion measures [50, 51].

##### Sleep quality

The 8-item PROMIS Pediatric Sleep Disturbance Short Form 8a will be used to assess sleep quality [52, 53]. It assesses adolescents’ experiences of sleep disturbances, including difficulties such as falling asleep and frequent awakenings. The questionnaire has demonstrated good content and construct validity among adolescents [52, 53].

##### Problematic smartphone use

The Smartphone addiction scale short version will be used to assess problematic smartphone use [54]. The Smartphone addiction scale short version consists of 10 items, assessing symptoms related to smartphone addiction such as daily-life disturbance, withdrawal, tolerance, and preoccupation. The scale has demonstrated excellent internal consistency and strong concurrent validity through significant correlations with related measures in adolescents [54].

#### Daily reports

To gain a more in-depth insight into participants’ habitual rhythm, emotional states, and social interactions throughout the study period, participants will receive a text message, where they will be reminded to complete the following 11 questions daily from baseline (day 1) to post intervention (day 42) (Supplementary Material 1). Six questions will ask them about their wake-up time and yesterday’s bedtime, school and/or work hours. Three questions will assess their current mood, perceived stress and sleepiness. Each item will be presented as a visual analogue scale (VAS), where participants place a marker along a continuum anchored by two descriptive words. VAS is chosen because it is quick to complete, impose minimal burden, and is sensitive to detecting small day-to-day fluctuations in emotional states and sleepiness. Prior research indicates that ecological momentary assessments with VAS can provide valid and reliable data on daily mood states in adolescents [55, 56]. The final two questions will assess if participants have had social interactions with individuals outside their household and who are not a part of their peer group. The 11 questions will be delivered via the SDU Connect application, which allows for momentary reporting in naturalistic settings.

#### Objective assessments

The following objective measures will be collected from day 1 until the post-intervention assessment (day 42), and some of the software-based assessments will continue to be collected until the long-term follow-up (day 70) (Fig. 2).

##### Physical activity, sleep, and stress

Participants will wear a Garmin Venu 4 watch capturing high resolution data from several sensors (e.g. accelerometry, thermometer, photoplethysmography) of circadian patterns of physical activity level and intensity, total sleep time and sleep stages, and heart rate variability. Garmin wearable devices with optical photoplethysmography have previously shown acceptable validity for several relevant outcomes, including selected measures of sleep, heart rate, and physical activity [57], and the validity of the specific device used in this trial will be evaluated separately in a dedicated validation study. The SDU Connect application will be used to synchronize data directly from the Garmin watch to a secure server at Avicenna Research using Garmin Health Software Developer Kit.

##### Social interactions

A bluetooth low energy (BLE) beacon (a small device that periodically transmits a unique identifier via BLE signals) will be attached to the Garmin Venu 4 watch. The SDU Connect application will scan the environment for 60 s every 5 min and record instances where participants from the same peer group’s BLE beacon are within a specified proximity range (approximately 5 m, Tx power − 20 dBm). Logging both the frequency and duration of these proximity events enables collection of time-stamped data on all social interactions between members of each peer group. This method has previously been used to track interaction between parent-adolescent dyads [58]. BLE beacons will also be set up in the participants own room and in the common room/living room of the participants home (if the participant is living together with other people e.g. family, friends, partner). This will enable objective tracking of time the participants spend in areas of the home where they are more likely to interact with other people.

In addition, we will track the geolocation of each participant’s smartphone in approximately 5-minute intervals via the SDU Connect application. Linking beacon proximity events with geolocation data will enable the identification of common locations and environments where each peer group typically meets at different times, thereby adding context to their social interactions. Moreover, it will be possible to assess the time participants spends outside of their home.

##### Screen media use

Screen media use will be tracked using three different methods. The SDU Connect application will be used to track smartphone and tablet use (overall screen time, time spent on specific applications, phone log, message log, and notification log) from Android and iOS devices. Computer use will be tracked using Manictime software (FinKit, Slovenia). Manictime data (overall computer usage and time spent on specific applications (e.g. Microsoft Word, Google Chrome) and websites (e.g. www.youtube.com, www.netflix.com) will be stored directly on a local server at the University of Southern Denmark. An in-house developed TV monitor will be used to track TV use on participants’ own TVs and any shared TVs in the household if any [59]. TV monitors will be equipped with a BLE beacon tracking when the participant is close to the TV (approximately 5 m, Tx power − 20 dBm). Combining use data from the TV monitor with BLE beacon data enables construction of time series datasets showing when the TV is on and a participant is nearby - indicating that they are likely watching.

#### Evaluation of the intervention

##### Questionnaire

Participants in the intervention group will complete a post-intervention questionnaire assessing their experiences with the 4-week screen media reduction. The questionnaire includes brief closed-ended and open-ended items designed to evaluate overall acceptability, perceived difficulty, and experienced impact of the intervention components (Supplementary Material 2).

##### Interview

The aim of the qualitative data collection is to explore how participants interpret and reflect on the screen media reduction, and how they perceive its influence, e.g. on their social lives and well-being. This will also include attention to unintended intervention outcomes [60]. By illuminating participants’ meaning-making processes, this component will provide a deeper understanding of the study’s quantitative findings.

A subsample of the participants in the intervention group will be invited to participate in individual or focus-group interviews. Participants will be selected through purposive sampling and diversity within and across peer-groups, including variation in characteristics such as age and sex. Guided by the concept of information power [61], the aim is not to maximize sample size, but to generate rich, contextually grounded data and well-substantiated interpretations.

The interviews will preferably be conducted in person at the university or another place preferred by the participant. We will strive to create a safe and trusting environment. We use an interview guide with concrete and open-ended questions, employing creative prompts, and engaging in reflexive listening [62]. This approach is designed to ensure that young participants feel safe and comfortable during the interviews, while also securing the depth and quality of the data generated.

Prior to interviews, additional oral information will be provided. Participation in the interviews is voluntary; participants may skip questions, withdraw at any time, or choose not to elaborate. They will be reminded that there are no “right” or “wrong” responses. Sensitive topics may arise, in which case the interviewer will pause and, if needed, provide information on support services.

#### Financial reimbursement

Participants in the PeerScreen trial will receive 1500 DKK (approximately 200 EUR) as taxable reimbursement for their participation in the study. This amount is provided solely as compensation for the time and inconvenience associated with the project.

### Data management and safety

The processing of personal data in this project will fully comply with the General Data Protection Regulation (GDPR) as well as the Danish Data Protection Act. The project is registered with the University of Southern Denmark’s internal record of research projects and thereby reported to the Danish Data Protection Agency through University of Southern Denmark’s Research & Innovation Organization (RIO), in accordance with national requirements. Data will be stored on secure platforms and used exclusively for research purposes.

### Statistical methods

#### Sample size calculations

Power calculations were conducted to ensure sufficient statistical power to detect clinically and scientifically meaningful difference on the WEMWBS score (the primary outcome). To achieve 80% power (alpha = 0.05), calculations were based on the following assumptions: A peer-group cluster size of 2 to 6 participants (mean = 3), a cluster size coefficient of variation of 0.2, an intracluster correlation coefficient of 0.05, a repeated measures correlation of 0.7, and a standard deviation of 9.8 points. Adjusting for our estimated cluster sizes, the target will be able to detect minimal mean differences of 3 points in WEMWBS. These assumptions indicated that 33 clusters per arm (198 participants) would be required. To account for a potential 10% rate of missing data or drop-out, the recruitment target was set at 80 clusters, corresponding to approximately 240 participants. With this sample size, the trial is powered at 80% to detect a minimal detectable mean difference of 2.85 points between groups (α = 0.05). Previous work suggests that 3 points on the WEMWBS represent the lower bound of meaningful change on the scale [37, 63].

#### Statistical analysis plan

A detailed statistical analysis plan describing the prespecified analyses will be finalized and published at clinicaltrials.gov before the final peer group of participants completes their final assessments.

##### Primary outcome

The primary outcome is mental wellbeing assessed using the WEMWBS scale. The primary endpoint is the change in mental wellbeing (WEMWBS-score) from baseline (Day 1) to Day 42.

Exploratory subgroup analyses will be conducted for the primary endpoint for the distribution of sex in the peer-groups (A: males only B: females only C: mixed peer groups) and mean age of the peer-group (A: <18 years B: ≥ 18 years). Details of preplanned sub-group analyses will be outlined in the statistical analysis plan.

##### Key confirmatory secondary outcomes

The following four key secondary endpoints are specified a priori to support mechanistic interpretation by quantifying behavioral changes plausibly linking reduced screen media use to changes in mental wellbeing:

- Face-to-face social interaction with peers (objective, Bluetooth beacons).

The primary peer-interaction metric will be total time spent in proximity-events with peer-group members (minutes/week) based on BLE beacon data collected via the SDU Connect application, which logs frequency and duration of proximity events within a predefined distance threshold. The exact aggregation will be prespecified in the statistical analysis plan.

- Face-to-face social interaction with other people (daily self-report).

Self-reported daily social interactions with people who are not a part of the peer group or the participants’ household (e.g. family members not living in the household, other friends, other relations). The exact aggregation of the self-reported interaction data will be prespecified in the statistical analysis plan.

- Total sleep time (objective, Garmin).

Total sleep time derived from continuous monitoring using Garmin Venu 4, as per device algorithms for total sleep time.

- Physical activity (objective, Garmin).

Physical activity derived from continuous monitoring using Garmin Venu 4. The exact algorithm for processing raw Garmin data will be prespecified in the statistical analysis plan.

## Discussion

### Methodological justification

This trial will address several caveats in the exiting research on the effects of reducing screen media use on mental and physical health outcomes among young people.

Several trials, where individuals are randomized to either reduce smartphone use or carry on as usual, have examined the effects on mental health, however, results are still inconclusive [14–16]. Additionally, reducing social media has been studied in many trials where participants are randomized on an individual level, and results points towards a positive effect of reducing social media use [10, 11]. However, individual level randomization may not be ecologically valid if the aim is to examine the effects of a broader societal reduction in screen media use. Moreover, previous interventions have generally targeted only one dimension of screen use at a time, rather than multiple potentially problematic screen-related behaviors in combination. This may be important, as screen-related effects on mental health may operate through several interrelated mechanisms, including plausible displacement of face-to-face social interaction, sleep disruption, and reduced physical activity, and exposure to social media content that may influence emotions, self-perceptions, and social experiences. In addition, many previous trials have been short-term, and relatively few have included participants younger than 18 years. Thus, one of the main advances of this trial is the group-based intervention, where peer-groups are randomly allocated to reduce their screen media use together or carry on with their usual screen media behaviors. A family-based randomization has previously shown positive effects on mental health for both children and their parents [64, 65], but to our knowledge no such interventions have been conducted among peer groups of adolescents and young adults.

The comprehensive objective assessment of screen media use in this trial enables collection of time-stamped data for screen media use on app-level for smartphones, tablets and computers. The detailed level of the data will allow us to get a deeper understanding of adolescents and young adults’ screen media use, and the patterns of their engagement with these devices, when they are asked to reduce their screen media use for 4 weeks. These data also provide an opportunity to explore, in a more data-driven and exploratory manner, which patterns of screen media use appear to be associated with the largest or smallest changes in mental well-being and the other outcomes obtained. In addition, the combination of objective time-stamped data for screen media use, and several behavioral measures (social interactions, physical activity, sleep and heart rate variability) enables a novel detailed investigation of the behavioral patterns within peer groups of young people and how reducing screen media use may affect peer-group dynamics. Furthermore, ensuring daily reports for mood, stress, and sleepiness over the longer term supports assessment of how participants respond to the intervention throughout the intervention period. This is particularly relevant given that the first few days of the intervention might be difficult for participants, akin to ‘craving’ this screen media use [66].

Finally, the qualitative data collected in this trial will aid a better understanding of how young people experience changes in their screen media behaviors and how it may influence their social lives and well-being. This type of knowledge has been lacking in previous trials on reduced screen media use, which have primarily focused on questionnaire-based outcomes and paid limited attention to participants’ lived experiences and experiences with such interventions, which could potentially clarify the processes through which reduced screen use may influence well-being.

### Anticipated limitations

The duration of the intervention (4 weeks) is still short, but as our aim is to achieve high compliance, 4 weeks were deemed feasible and acceptable based on three interviews with groups of adolescents and young adults conducted during the intervention development. This expectation is further supported by findings from a two-week family-based screen media reduction, in which high compliance was achieved despite participants being allowed only 3 h of screen media use per week [67], whereas participants in the current study are allowed up to 21 h per week. Although, we aim to achieve high compliance, there are also common challenges in behavioral interventions targeting screen media behaviors that we cannot overcome. First, participants may bypass the monitoring system by using other non-tracked devices, meaning they may appear compliant in our tracking datasets, but in reality, they were non-complaint. Also, due to the behavioral nature of the intervention, participants cannot be blinded to their group allocation, which may result in bias for the self-reported outcome measures and emphasizes the importance of the planned objective measures.

### Ethical considerations

Gaining knowledge on the potential effects of screen media use in 13–24 years-old, is necessary because screen media behaviors undergo substantial changes with age. It is particularly important to include adolescents aged 13–18 years as experimental evidence on the effects of reducing screen media use in this population remains very limited. Adolescents are often granted greater autonomy regarding screen media use around the age of 13, with fewer parental rules governing their use [68]. This transition may increase the risk of developing unhealthy screen media habits. Additionally, some research suggests that adolescents may be particularly sensitive to the negative effects of social media use around the ages of 13–15 years [69]. There are no known risks associated with reducing screen media use for a period of 4 weeks.

Our comprehensive tracking protocol may raise privacy concerns for some participants. However, no personal content is collected or shared with the research team. We track only metadata such as time spent on different devices (smartphone, tablet, computer, and television), applications (e.g., TikTok), and websites (e.g., Netflix.com), but not the specific content participants consume (e.g., videos, posts, or movies). Participants are thoroughly informed about the data collection procedures, and all data are stored on secure servers in compliance with the GDPR.

### Implications

The PeerScreen trial will contribute important experimental evidence on the causal effects of recreational screen media use on the well-being and related outcomes of young people. It will contribute to a more fundamental understanding of whether, and through which behavioral pathways, recreational screen media use influences adolescents and young adults. Such evidence may inform public health recommendations, policies, and prevention strategies aimed at promoting healthier habits among adolescents and young adults. By intervening at the level of naturally occurring peer groups, the trial may also provide important insights into how peer-group social dynamics may influence the effect of interventions aimed at reducing screen media use.

## Supplementary Information

Supplementary Material 1.

Supplementary Material 2.

Supplementary Material 3.

## Data Availability

No datasets were generated or analysed during the current study.

## Abbreviations

ANS: Autonomic Nervous System
BLE: Bluetooth Low Energy
GAD-2: Generalized Anxiety Disorder 2-item
GDPR: General Data Protection Regulation
VAS: Visual Analog Scale
WEMWBS: Warwick-Edinburgh Mental Wellbeing Scale
