# Supplementary material for: Effects of reducing screen media use on mental health, sleep, physical activity, heart rate variability, and behavioral patterns in peergroups of young people: a study protocol for a cluster-randomized controlled trial
Source: BMC Psychol. 2026 May 28;14:1098. doi: 10.1186/s40359-026-04827-5 (PMC13397752; doi:10.1186/s40359-026-04827-5)
Supplement: Supplementary file 1 — Supplementary Material 1. [file 40359_2026_4827_MOESM1_ESM.docx]

**Daily reports**

**How is your mood right now?**
Please indicate your mood on the scale below.

Very good ------------------------------------------------ Very bad

**How tired do you feel right now?**
Please indicate your mood on the scale below.

Very tired ----------------------------------------------- Not tired at all

**How stressed do you feel right now?**
Please indicate your mood on the scale below.

Very stressed ----------------------------------------------- Not stressed at all

Think about yesterday.

**During your leisure time yesterday, were you together in person with people you do not live with and who are not part of your friend group?**
Leisure time = time outside of school and work

- Yes
- No

If ”Yes”: **Which people were you together with?**
You may select more than one option.

- Family members you do not live with
- Boyfriend/girlfriend or partner you do not live with
- Friends you do not live with and who are not part of your friend group
- Acquaintances from e.g. clubs or leisure activities, whom you do not live with
- Other acquaintances you do not live with

**What time did you start school/education yesterday?**
[Dropdown with timeslots]

**What time did you leave school/education yesterday?**
[Dropdown with timeslots]

**What time did you start work yesterday?**
[Dropdown with timeslots]

**What time did you leave work yesterday?**
[Dropdown with timeslots]

**What time did you go to bed yesterday?**
[Dropdown with timeslots]

**What time did you wake up today?**
[Dropdown with timeslots]
