# Supplementary material for: Effects of reducing screen media use on mental health, sleep, physical activity, heart rate variability, and behavioral patterns in peergroups of young people: a study protocol for a cluster-randomized controlled trial
Source: BMC Psychol. 2026 May 28;14:1098. doi: 10.1186/s40359-026-04827-5 (PMC13397752; doi:10.1186/s40359-026-04827-5)
Supplement: Supplementary file 2 — Supplementary Material 2. [file 40359_2026_4827_MOESM2_ESM.docx]

**Questionnaire – Evaluation of the intervention**

**How was your overall experience with the screen reduction over the past 4 weeks?**

- Very positive
- Mostly positive
- Neither positive nor negative
- Mostly negative
- Very negative

Please feel free to elaborate on your answer: ____________________________________________

**How difficult was it to comply with the screen media reduction?**

- Very difficult
- Difficult
- Neither difficult nor easy
- Easy
- Very easy

Please feel free to elaborate on your answer: ____________________________________________

**To what degree did you experience that carrying out the screen reduction together with some of your friends was helpful?**

- To a very high degree
- To a high degree
- To some degree
- To a lesser degree
- Not at all

Please feel free to elaborate on your answer: ____________________________________________

**How was your experience of having only 1 hour per day to use your smartphone?**

Write your answer here: _______________________________________________

**How was your experience of having only 14 hours per week for computer, TV, and tablet use?**

Write your answer here: _______________________________________________

**How was your experience of not having screen media devices in your bedroom at night?**

Write your answer here: _______________________________________________

**What did you spend your time doing instead of using your smartphone?**

Write your answer here: _______________________________________________

**Did you feel that 1 hour per day was enough to handle necessary tasks on your smartphone?**

- Yes
- No

**If “No”: How much smartphone time do you realistically think you need per day?**

- 1.5 hours
- 2 hours
- 2.5 hours
- 3 hours
- 3.5 hours
- 4 hours
- 4.5 hours
- More than 5 hours

Please feel free to elaborate on your answer: ____________________________________________

After participating, I will continue to try to limit my screen media use.

- **Yes, please elaborate on your answer: ___________________________________________**
- **No, please elaborate on your answer:** ____________________________________________

**Did you experience that the screen media reduction affected your social life (positively or negatively)?**

- **Yes, please elaborate on your answer: ___________________________________________**
- **No, please elaborate on your answer:** ____________________________________________

**Did you experience that the screen media reduction affected your well-being (positively or negatively)?**

- **Yes, please elaborate on your answer: ___________________________________________**
- **No, please elaborate on your answer:** ____________________________________________

**Did you experience any other changes in connection with the screen media reduction (positive or negative)?**

- **Yes, please elaborate on your answer: ___________________________________________**
- **No, please elaborate on your answer:** ____________________________________________

**What do you think did not work well during the screen media reduction period?**

Write your answer here: _______________________________________________

**What do you think was good about the screen media reduction period?**

Write your answer here: _______________________________________________

**Is there anything from the screen media reduction period that made an impression on you and that you will take with you going forward?**

Write your answer here: _______________________________________________
